# Medication adherence and its associated factors among diabetic patients at Zewditu Memorial Hospital, Addis Ababa, Ethiopia

**DOI:** 10.1186/s13104-017-3025-7

**Published:** 2017-12-04

**Authors:** Muhammed Ali, Tigestu Alemu, Oumer Sada

**Affiliations:** Department of Pharmacology and Clinical Pharmacy, School of Pharmacy, College of Health Sciences, Addis Ababa University, Addis Ababa, Ethiopia

**Keywords:** Addis Ababa, Adherence, Anti diabetic medication, Diabetes, Zewditu Memorial Hospital

## Abstract

**Objective:**

Diabetes is a global problem with devastating human, social and economic impact. Anti-diabetic medications play a major role in the glycemic control of patients with diabetes. However, inadequate adherence compromises safety and treatment effectiveness, leading to increased mortality and morbidity. The aim of this study was to assess adherence to anti-diabetic medications and associated factors among patient with diabetes mellitus receiving care at Zewditu Memorial Hospital.

**Results:**

Among the total of 146 diabetic patients (mean age 46.5 ± 14.7), the level of adherence to anti diabetic medication was 54.8% (80) whilst 45.2% (66) of the participants were non adherent. Multiple logistic regression showed that knowledge of medication (AOR = 4.905, 95% CI 1.64–14.62, medication availability (AOR = 0.175, 95% CI 0.031–0.987) and education level (AOR = 13.65, 95% CI 1.45–128.456) were reasons for non-adherence.

## Introduction

Diabetes mellitus (DM) is probably one of the oldest diseases known to man. It is defined as a “metabolic disorder caused by different factors characterized by a chronic high level of blood sugar with disturbances to carbohydrate, fat, and protein metabolism resulting from defects in insulin secretion, insulin action, or both. It is a complex, chronic diseases requiring continuous medical care with multifactorial risk-reduction strategies beyond glycemic control [1, 2].

The 2014 report stated that the global prevalence of diabetes among adults was 422 million, compared to 108 million in 1980. This prevalence is nearly doubled since 1980, rising from 4.7 to 8.5% in the adult population. By 2030 this would have risen to 552 million [3, 4].

According to IDF 2003, diabetes is a global problem with devastating human, social and economic impact. Globally DM is the fifth leading cause of death by disease [5]. Every 7 s, diabetes causes the death of an individual worldwide, and in 2014 alone, 4.9 million deaths were attributed to diabetes with 80% of deaths related to diabetes reported from low- and middle-income countries [6]. According to WHO, diabetes was once considered a rare disease in sub-Saharan Africa. But in 2010, over 12 million people in sub-Saharan Africa are estimated to have diabetes, and 330,000 people died from diabetes-related conditions. Over the next 20 years, it is predicted that sub-Saharan Africa will have the highest growth in the number of people with diabetes of any region in the world—the 2010 estimated number is predicted to almost double in 20 years, reaching 23.9 million by 2030 [7].

Ethiopia is estimated to have around 1.5 million people with diabetes. According to WHO data published in 2011 DM dates in Ethiopia reached 21,550 or 2.62% of total deaths. The age adjusted death rate is 61.96 per 100,000 of population ranks Ethiopia number 28 in the world and it is the 7th leading cause of death in Ethiopia [8].

A latest WHO report indicates that because the magnitude of non-adherence and the scope of its sequelae are so alarming, more health benefits worldwide could be achieved by improving adherence to available medications than by developing new treatment approaches. Studies revealed that compliance to chronic medications in high income countries is 50%. In low income countries, the prevalence is even lower [9]. Poor compliance complicates the challenges of improving health in developing countries, and leads to waste and underutilization of already limited resources. Compliance studies are peculiar to every community and culture.

Adherence to medication is influenced by several factors such as lack of information, complexity of regimen, concomitant disease, and perceptions of benefit, side effects, medication cost, long duration and emotional wellbeing. Personality and cultural factors may influence adherence-compliance rates. Institutional factors such as the availability of medication at the hospital pharmacy, cost of medications, prescription patterns and accessibility also affects adherence. Again the personal beliefs, knowledge on disease and medication, forgetfulness and financial burden also reduce adherence level. These factors are interrelated.

This study was aimed to assess level of adherence and identify factors that are particular to diabetic patients who receive care at Zewditu Memorial Hospital.

## Main text

### Methods

#### Study area and period

Prospective cross sectional study was conducted at chronic follow up unit of Zewditu Memorial Hospital. It is a general hospital in central Addis Ababa. The hospital deals also with palliative care, HIV counseling and testing, and post-exposure prophylaxis (PEP) service. There are different clinics for follow-up of patients with chronic illness at the hospital. The study was conducted from February 20, 2017 to April 20, 2017.

#### Study participants and sample size determination

The study participants were all patients with diabetes, aged at least 18 years, attending the diabetic clinic, which have been on anti-diabetic medication for greater than 6 months and gave informed consent to participate in the study. We excluded those who were very ill and those who were newly diagnosed with diabetes and on medication for less than 6 month from the study. A total of 146 participants were included by using single proportion formula and using p value of 0.89 from previous study and correction formula. The first patient was selected randomly and every 7th patient was included in the study. The dependent variable was adherence to anti diabetic medication.

#### Data collection procedures

Trained data collectors used pretested structured questionnaires to collect information on: patient demographics and variables used to assess patient adherence. And the data was cleared and checked every day for completeness and consistency. The level of adherence assessment was measured by Morisky Medication Adherence Scale-8 (MMAS-8).

#### Operational definitions

*Adherence* The extent to which a person’s behavior—taking medication, following a diet, and/or executing lifestyle changes—corresponds with agreed recommendations from a health care provider.

*Adherent* Patients who score 0 based on Morisky Medication Adherence Scale-8 (MMAS-8).

*Non adherent* Patients who score 1–8 based on Morisky Medication Adherence Scale-8 (MMAS-8).

#### Data management and analysis

The collected data were sorted, coded and entered into SPSS version 20 software for analysis. Descriptive statics was generated to summarize patient socio-demographic data; bivariate analysis was implemented to identify associated factors. The crude odds ratio (COR), adjusted odds ratio (AOR) and 95% confidence interval (CI) were performed to determine factors associated with anti-diabetic medications adherence and a p value of 0.05 or less was considered statistically significant.

#### Ethical consideration

Approval request paper cleared by ethical review committee from Addis Ababa University, College of health science, and school of pharmacy was submitted to Zewditu Memorial Hospital to undertake the study. Written informed consent was obtained from study participants. Confidentiality was maintained throughout the study process.

### Results

#### Socio-demographic characteristics of participants

A total of 146 diabetics aged 18–79 were recruited. All the participants had been on diabetic medication for 6 months and above. The mean age of the participant was 46.5 years (± 14.6) with a range of (18–79 years). The majority of the participants were above 50 years. About 23.3% (34) of the participants aged 51–60 years and 17.8% (26/146) were older than 60 years. Female participants formed the majority.

There were 54.1% (79) females and 45.9% (67) males. Most of participants were married. The married participants were 69.2% (101). For educational level, 34.9% (51) of the participant reported primary education as their highest form of education. However, 223.3% (34) reported that they have had no formal education. Sixty-five (44.5%) participants were unemployed and 44 (30.1%) were self-employed. Table 1 shows socio demographic characteristics of the participant.

**Table 1:** Socio-demographic characteristics of patient with diabetes mellitus attending diabetic clinic of Zewuditu Memorial Hospital

Variable	Frequency	Percent
Sex		
Male	67	45.9
Female	79	54.1
Age		
18–30	25	17.1
31–40	31	21.2
41–50	30	20.5
51–60	34	23.3
> 60	26	17.8
Religion		
Orthodox	58	29.5
Muslim	43	39.7
Protestant	26	17.8
Catholic	19	13.0
Ethnicity		
Amhara	57	39.0
Oromo	41	28.1
Tigre	22	15.1
Other	26	17.8
Marital status		
Single	23	15.8
Married	101	69.2
Divorced	11	7.5
Widowed	11	7.5
Educational level		
None	34	23.3
Primary level	51	34.9
Secondary level	43	29.5
Tertiary level	18	12.3
Employment		
Unemployment	65	44.5
Self employed	44	30.1
Government	18	12.3
Private	19	13.0
Monthly income in birr		
Low income (< 1000)	77	52.7
Middle income (1001–2000)	33	22.6
High income (2001–3000)	26	17.8
Very high income (> 3000)	10	6.8

#### Clinical characteristics of participants

Type 2 diabetics the commonest type of diabetics, 54% (80) participant had been diagnosed with type 2 diabetics. However 25.3% (37) of the participants did not know type of diabetics. The mean number of medication prescribed was 1.51 (± 0.816).

#### Institutional factors

More than 20% (35) of the respondents did not have all their diabetic medication available at the hospital pharmacy. About 71.2% (104) reported that all their diabetic medication was covered by health insurance. Majority of the participant, 97.3% (142) reported that there was not any difficulty in getting their prescribed medication.

Regarding the service rendered, a larger proportion 74.7% (110) of the participants reported that they were satisfied with it.

#### Participants knowledge of diabetes and medication

**Fig. 1:**
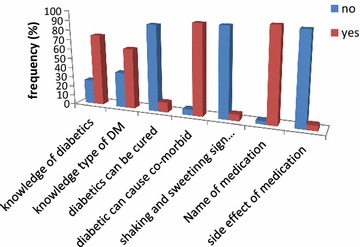
Distribution of participants on knowledge of diabetics and medication

About 96.6% (141) reported they knew the name of their medication. However 93.8% (137) reported that they do not know any side effect of their diabetic medication. Majority of the participants had knowledge on diabetes mellitus, types, and sign of hypoglycemia and cause co-morbid disease. However 10.3% (15) reported that diabetics can be cured (Fig. 1).

#### Determining the level of adherence

The level of adherence was assessed using eight item-Morisky Medication Adherence Scale (MMAS-8). Participants who had score of 0 were classified to adherent. The minimum score was 0 and the maximum score 4. Based on this classification 54.8% (80) of the participants were adherent whilst 45.2% (66) of the participants were non adherent.

#### Factor affecting adherence

*On bivariate analysis* Medication availability, complexity of regimen, satisfaction and Education level were found to be significantly associated. On the other hand, multiple logistic regression showed that knowledge of medication (AOR = 4.905, 95% CI 1.64–14.62, medication availability (AOR = 0.175, 95% CI 0.031–0.987) and Education level (AOR = 13.65, 95% CI 1.45–128.456) were reasons for non-adherence (Table 2).

**Table 2:** Factors affecting medication adherence of patients attending Zewuditu Memorial Hospital

Variable	COR [95% CI]	AOR [95% CI]	p value
Knowledge of medication	0.179 (0.028–1.156)	4.905 (1.64–14.62)	*0.004*
Medication availability	0.011 (0.001–0.086)	0.175 (0.031–0.987)	*0.048*
Satisfaction	0.012 (0.002–0.971)	1.78 (0.518–6.128)	0.36
Perception about treatment outcome	2.089 (0.525–8.315)	1.955 (0.453–8.44)	0.369
Side effect knowledge	0.475 (0.109–2.068)	0.355 (0.064–1.956)	0.234
Complexity of regiment	31.952 (9.12–11.84)	2.822 (0.786–10.23)	0.112
Length of treatment	1.545 (0.165–14.47)	0.534 (.054–4.992)	0.465
Education level	0.027 (0.003–0.218)	13.65 (1.45–128.456	*0.022*

p value of 0.05 or less was considered statistically significant (in italics)

### Discussion

Currently, there is no single measure accepted as the gold standard to measure medication adherence since all commonly employed methods have drawbacks. In this study, from the available methods, a self-reported 8-item Morisky Medication Adherence Scale was used to assess medication non-adherence.

A systematic review on the adherence to medication among diabetic patients showed that the average compliance to the oral hypoglycemic agents ranged from 36 to 93% [10].

The magnitude of adherence level in this study was 54.8%. In contrast to this finding, two studies in Ethiopia showed that the patients self-reported adherence rate to anti-diabetic medication was 72.2 and 68.8% [11, 12]. The difference may be attributed to, methodological variations and ways of measurement for adherence level.

Health care providers should be cognizant of knowledge of patients on diabetes and how this may affect long-term efforts to successful management of diabetes mellitus. Emphasis on awareness creation about diabetes and its management is important to achieve positive diabetes outcomes. It was revealed in this study that patients who had no knowledge about diabetes were five times more likely to be non-adherent as compared to patients who had knowledge on diabetes. Other studies are also consistent with this finding.

Regarding educational level, it was found to be significantly associated with the level of adherence to the treatment regimen. From different studies education has been identified as major socioeconomic determinant of adherence to anti-diabetic medication. Low educational level has been associated with higher rates of non-adherence. This was supported by previous researches done in UAE [13]. Being illiterate makes learning more difficult; as diabetes drug therapy gets more complex, patients are required to have more complex cognitive skills to be able to understand the prescribed drug therapy and to adhere to treatment for good glucose control.

Availability of medications was one of the variables that found to be significantly associated with the adherence status of the respondents. Findings of this study collaborated with other studies indicated an association between medication adherence and medication availability [11, 13]. Unavailability of medications in the health institution has negative impact on patient adherence, especially when it is accompanied by low economic status. Because the patient cannot afford to bay medication from the private sectors, where medications are usually costly.

Concerning institutional factors, number of medication prescribed was independently associated with non-adherence. Participants who had complex regimen were more likely to have poor adherence. This finding is similar to study done in Jimma University Specialized Hospital [14]. This can be explained by the perception that participants who had more medications perceived themselves as severely ill and hope less to cure from chronic disease. Another independently associated factor was patient satisfaction. This indicates that patients satisfied by service rendered in the hospital were adherent to their prescribed medication. Better relationship between health professionals and clients improve satisfaction. Findings from a study in Nigeria found an association between poor patient provider communication, lack of trust in the provider and adherence [15, 16].

### Conclusion

In general, level of adherence to prescribed medications was poor among diabetic patients in diabetic clinic of ZMH. Knowledge of medication, medication availability and education level were found to be factors contributory for medication non-adherence, all of which are modifiable factors. A lot should be done to maximize the medication adherence of these patients so that they can recognize the full importance of prescribed therapies.

### Limitation

The study is single centered and it may limit its generalizability.

## Abbreviations

MMAS-8: Adherence Scale-8
DM: diabetes mellitus
